# Bridging Neural Topology and Affective Computing: Graph Attention for EEG Emotion Recognition

**DOI:** 10.1007/s10916-026-02345-w

**Published:** 2026-02-20

**Authors:** Wenyang Yang, Jingrui Yuan, Bingnan Duan, Steven Kwok Keung Chow

**Affiliations:** 1 School of Computer Science, Xi’an Shiyou University, Xi’an, 710065 P.R. China; 2 Key Laboratory of Modern Teaching Technology, Ministry of Education, Shaanxi 710065 Xi’an, P.R. China; 3 Department of Medical Imaging and Radiation Sciences, School of Primary and Allied Health Care, Faculty of Medicine Nursing and Health Sciences, Monash University, Wellington Road, Clayton, VIC 3800 Australia

**Keywords:** EEG, Emotion recognition, Spatial attention, Graph neural network, Affective computing, Neurophysiological alignment

## Abstract

Electroencephalography (EEG) offers high temporal resolution and strong physiological validity for emotion recognition. However, complex spatial organization and inter-subject variability present major modeling challenges. Graph-based spatial attention mechanisms have emerged as a key solution, preserving brain topological priors while adaptively emphasizing emotion-relevant regions and connections. This review summarizes advances in graph convolutional networks (GCN) and graph attention networks (GAT), covering representative studies under both subject-dependent and subject-independent settings. In architectural innovations, this paper critically evaluates the implicit impact of experimental factors, including preprocessing pipelines and validation protocols, on performance, and proposes a standardized framework to enhance reproducibility. Existing research demonstrates progressive transitions from static to dynamic graphs and from single-domain to multimodal fusion guided by physiological priors. Future research is expected to focus on enhancing model efficiency, strengthening neurophysiological alignment, integrating multimodal information and enhancing subject-independent generalization, and extending applications to affective neuroscience and clinical contexts. These developments collectively drive EEG-based emotion recognition toward more efficient, interpretable, and translationally valuable affective computing systems.

## Introduction

Emotion is a complex psychological and physiological response to external stimuli, profoundly shaping cognition, decision-making, and behavior. With the rapid progress of artificial intelligence and brain-computer interface (BCI) technologies, emotion recognition has become a core research task with applications in human-computer interaction, mental health monitoring, adaptive learning, immersive entertainment, and intelligent driving [1].

Electroencephalography (EEG), which records central neural activity, is a key modality for emotion recognition due to its non-invasive, high temporal resolution, and cost-effectiveness [2]. In neuroscience and medical research, EEG-based emotion analysis has been widely used to identify emotional dysregulation in patients, evaluate the outcomes of therapeutic interventions, and provide objective neural indicators for early diagnosis and personalized treatment of affective disorders [3]. It also supports studies of emotion regulation mechanisms and serves as a reliable neurophysiological marker for mental health assessment [4]. Unlike external cues such as facial expressions or speech, EEG provides objective information less subject to voluntary control, making it particularly suited for detecting subtle, implicit, or concealed emotions [5]. However, EEG signals are high-dimensional, nonlinear, and low in signal-to-noise ratio, with pronounced inter-individual variability [6]. Moreover, emotion processing depends on coordinated activations and long-range interactions across brain regions, reflecting spatial dependencies that exceed the local modeling capacity of traditional convolutional and recurrent neural networks (CNNs and RNNs) [7, 8]. Capturing such complex cross-regional relationships remains a major challenge.

Graph neural networks (GNNs) provide a promising solution by leveraging the non-Euclidean structure of EEG electrodes, which aligns with functional brain networks [9, 10]. By treating channels as graph nodes and applying convolution, GNNs explicitly capture topological relationships and functional connectivity [11]. Attention mechanisms further enhance EEG emotion recognition by adaptively weighting channels, spatial locations, and frequency bands, emphasizing emotion-relevant features while suppressing noise [12]. The integration of attention mechanisms yields graph-based spatial attention, which combines GNN topological priors with dynamic dependency learning. The hybrid paradigm has shown improved discriminability, interpretability, and subject-independent robustness [13, 14].

In summary, graph-based spatial attention has become a key research direction in EEG emotion recognition. This paper reviews its recent advances, focusing on GCN- and GAT-based methods, representative improvements, and applications under various experimental settings. Finally, challenges in generalization, neural consistency, and lightweight implementation are discussed.

### Searching Method

A literature review was conducted in accordance with the PRISMA (Preferred Reporting Items for Systematic Reviews and Meta-Analyses) guidelines [15]. Scopus, Web of Science, Google Scholar, IEEE, PubMed, ACM, MEDLINE, and IJCAI were included in the search across eight major academic databases, yielding a total of 362 records and 193 unique publications after duplicate removal.

**Fig. 1:**
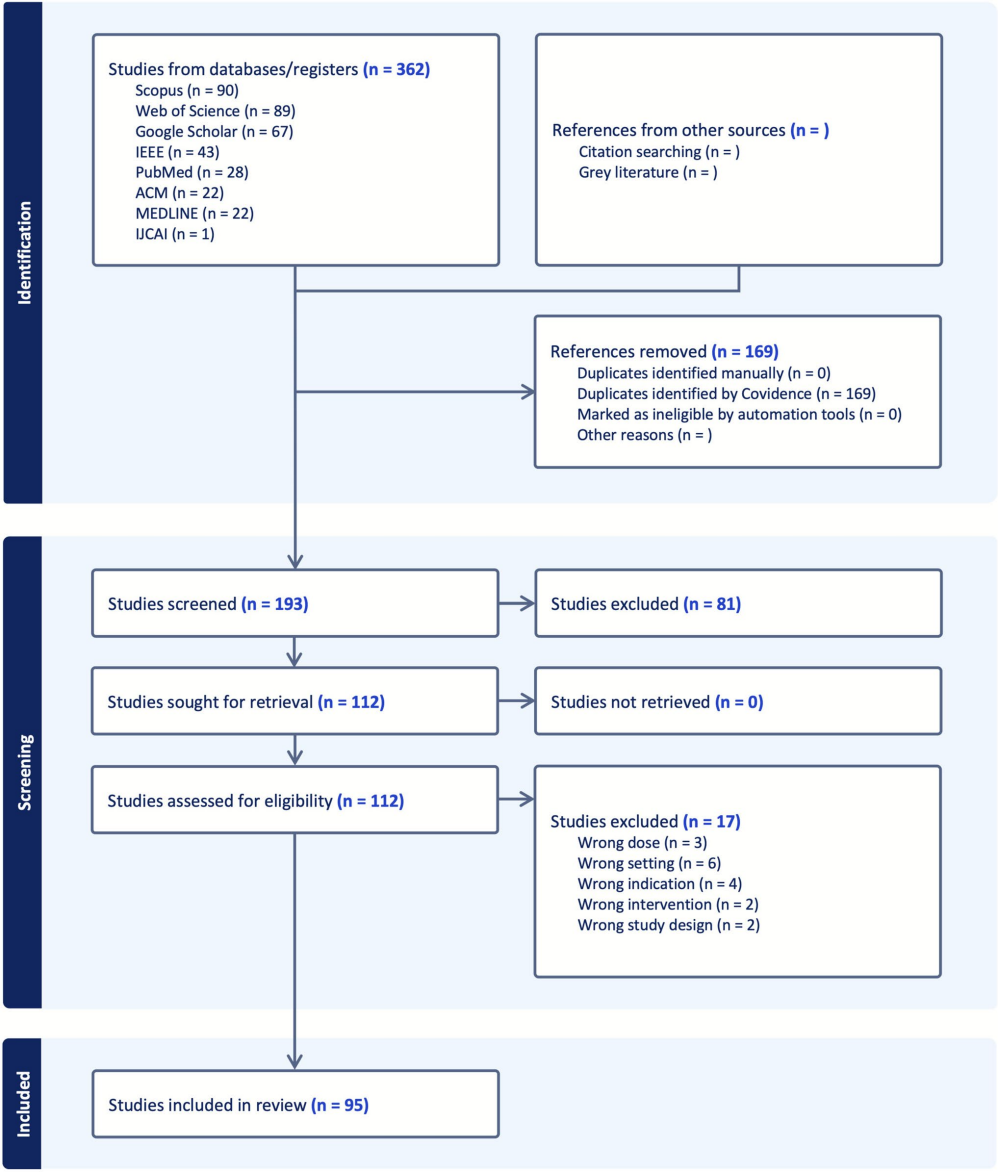
PRISMA Flowchart of Literature Screening Flow and Process on inclusion and exclusion of literatures

Figure 1 presents the literature screening process using a PRISMA flowchart, which outlines the quantitative reduction from initial retrieval to final inclusion. Duplicate removal using Covidence software excluded 169 records. In the first screening phase, based on titles and abstracts, 134 publications were eliminated due to irrelevant research contexts, unsuitable methodological approaches, or incompatible study designs. Full-text evaluations were then conducted for 59 articles, resulting in 34 studies that met the predefined inclusion criteria. The final selection comprised 34 high-quality studies that provide a solid foundation for analyzing EEG-based emotion recognition using spatial attention mechanisms, while minimizing potential selection bias.

## Foundations and Challenges of EEG-based Emotion Recognition

This section establishes the foundation for the subsequent systematic review of graph-based spatial attention mechanisms in EEG-based emotion recognition. The content progresses from theoretical emotion models to computational paradigms and finally to key challenges.

### Theoretical Models and Computational Paradigms

**Fig. 2:**
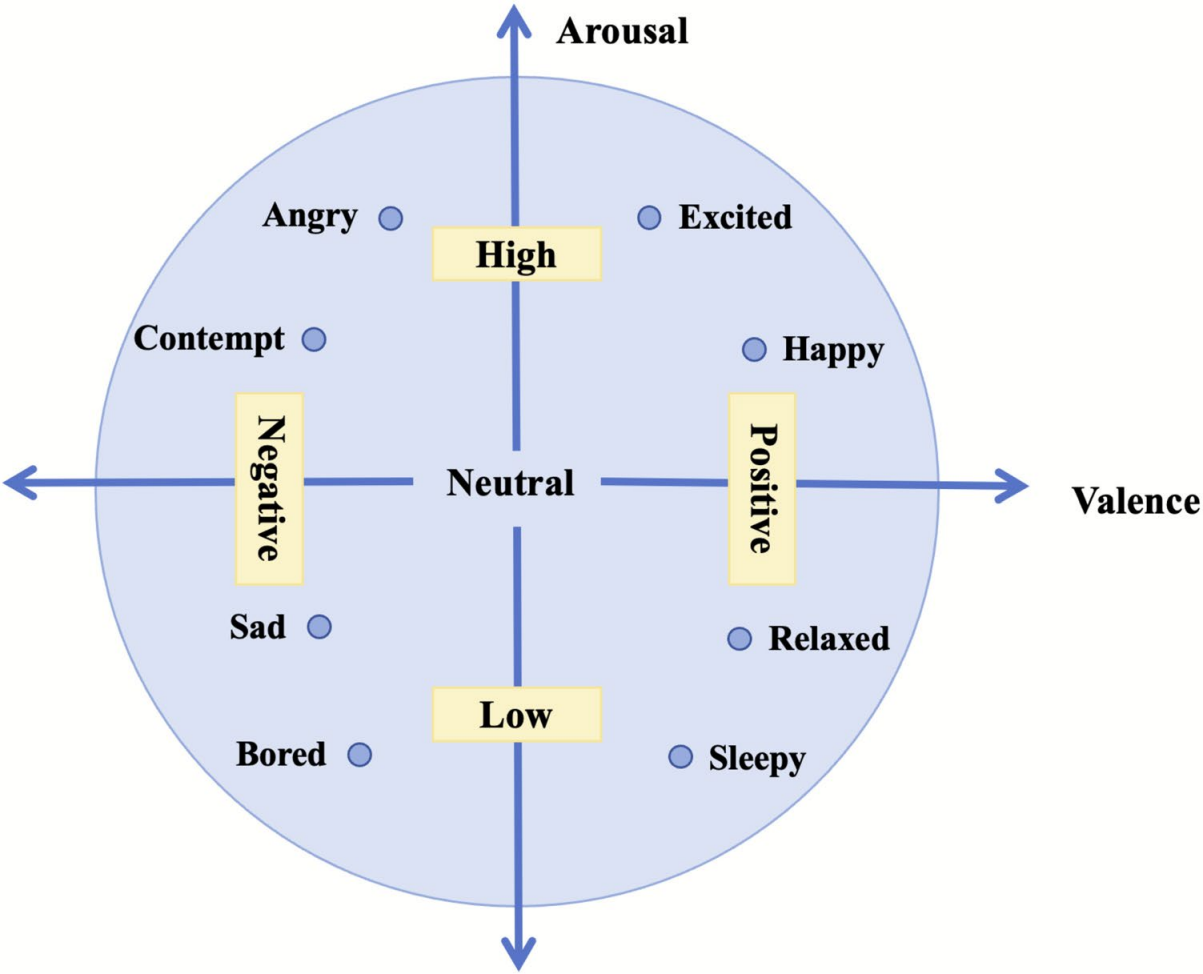
Figure of James Russell’s classification model for different affect emotions [19]

Accurate quantification and formal description of human emotions are prerequisites for constructing objective EEG-based emotion recognition systems. Theoretical models of emotion provide the conceptual foundation for understanding the intrinsic structure of affective states, guiding both the induction and annotation of emotions in EEG experiments and shaping subsequent feature extraction and machine learning design. Current studies on electroencephalography-based emotion recognition mainly adopt two paradigms, namely discrete emotion models and dimensional emotion models [16–18]. The discrete model, exemplified by Ekman’s theory of basic emotions, categorizes affective states into a finite set such as happiness, anger, and sadness, thereby supporting multi-class classification tasks [16]. Figure 2 shows Russell’s dimensional model of the two-dimensional valence-arousal space, which conceptualises emotions as coordinates in a continuous space, capturing the dynamic nature of affective processes more effectively [19].

At the computational level, such theoretical models are operationalized into standardized task paradigms. Tasks derived from the discrete model are formulated as multi-class classification, ranging from binary to multiple categories with corresponding increases in complexity [20]. Dimensional models are typically modeled as regression problems or discretized into quadrants on the valence–arousal plane to form four-class classification tasks [20]. For label acquisition, self-report methods, particularly the Self-Assessment Manikin scale, are widely adopted to ensure the reliability of training data. Thus, theoretical models and task paradigms jointly define the input–output structure of EEG-based emotion recognition. However, such definitions do not resolve the fundamental challenges, which primarily stem from the complex characteristics of EEG signals rather than from task formulation.

### Signal Characteristics and Intrinsic Challenges

Unlike event-related potentials elicited by external stimuli, the reflections of emotional states in electroencephalography are typically weaker, more dispersed, and more elusive [2, 21]. These characteristics introduce multiple challenges for emotion recognition from electroencephalography signals, which could be summarized in four main aspects.

First, high dimensionality combined with a low signal-to-noise ratio makes emotion-related signals highly susceptible to being obscured [6]. In contrast to the clear structure of event-related potentials, brain responses induced by endogenous emotions are considerably weaker. Moreover, physiological artifacts such as facial expressions or eye blinks could be strongly correlated with emotional states, further complicating the extraction of valid features [22].

Second, the non-stationarity and nonlinear nature of electroencephalography signals exacerbates modeling difficulties [23]. Emotions inherently involve dynamic processes including induction, maintenance, and attenuation. The natural temporal evolution endows the signals with pronounced nonstationary characteristics [24]. Consequently, models are required not only to recognize static emotional states but also to capture the transitions between dynamic affective conditions.

Third, substantial inter-individual variability magnifies the challenge of generalization. Even under identical stimuli, differences in response intensity, regulation strategies, and subjective labeling could emerge across individuals [25]. Such variability leads to highly individualized emotion-related patterns, making subject-independent generalization one of the most intractable issues in electroencephalography-based emotion recognition.

Finally, the functional organization of the brain exhibits non-Euclidean properties. Emotional processes are not determined by a single localized region but depend on large-scale distributed networks [26]. The spatial distribution of electroencephalography electrodes represents a projection of this topological structure, and emotional information is often encoded in complex spatiotemporal patterns across channels [27]. This implies that modeling approaches limited to single-channel temporal signals are insufficient for decoding emotional processes, highlighting the necessity of capturing spatial dependencies.

In summary, the main obstacles of electroencephalography-based emotion recognition arise not only from task definition but also from the signals, which exhibit intrinsic complexity. Such challenges have directly driven the research community to move from conventional classification models toward specialized spatial modeling strategies, establishing the logical foundation for the subsequent development of spatial attention mechanisms.

## Core Formulations of Spatial Attention Mechanisms

The section formalizes several representative attention mechanisms, including channel attention, multi-head attention, and graph-based spatial modeling. The first two provide the theoretical foundation for the latter.

### Channel Attention Mechanism

The channel attention mechanism adaptively assigns weights along the channel dimension, enabling the model to emphasize EEG channels most relevant to emotional processing. The core idea is to compute channel weights via global pooling and nonlinear transformations, then apply them to the original features to highlight informative signals while suppressing redundancy. Representative methods include the Squeeze-and-Excitation Net (SE-Net) [28] and the Convolutional Block Attention Module (CBAM) [29].

**Fig. 3:**
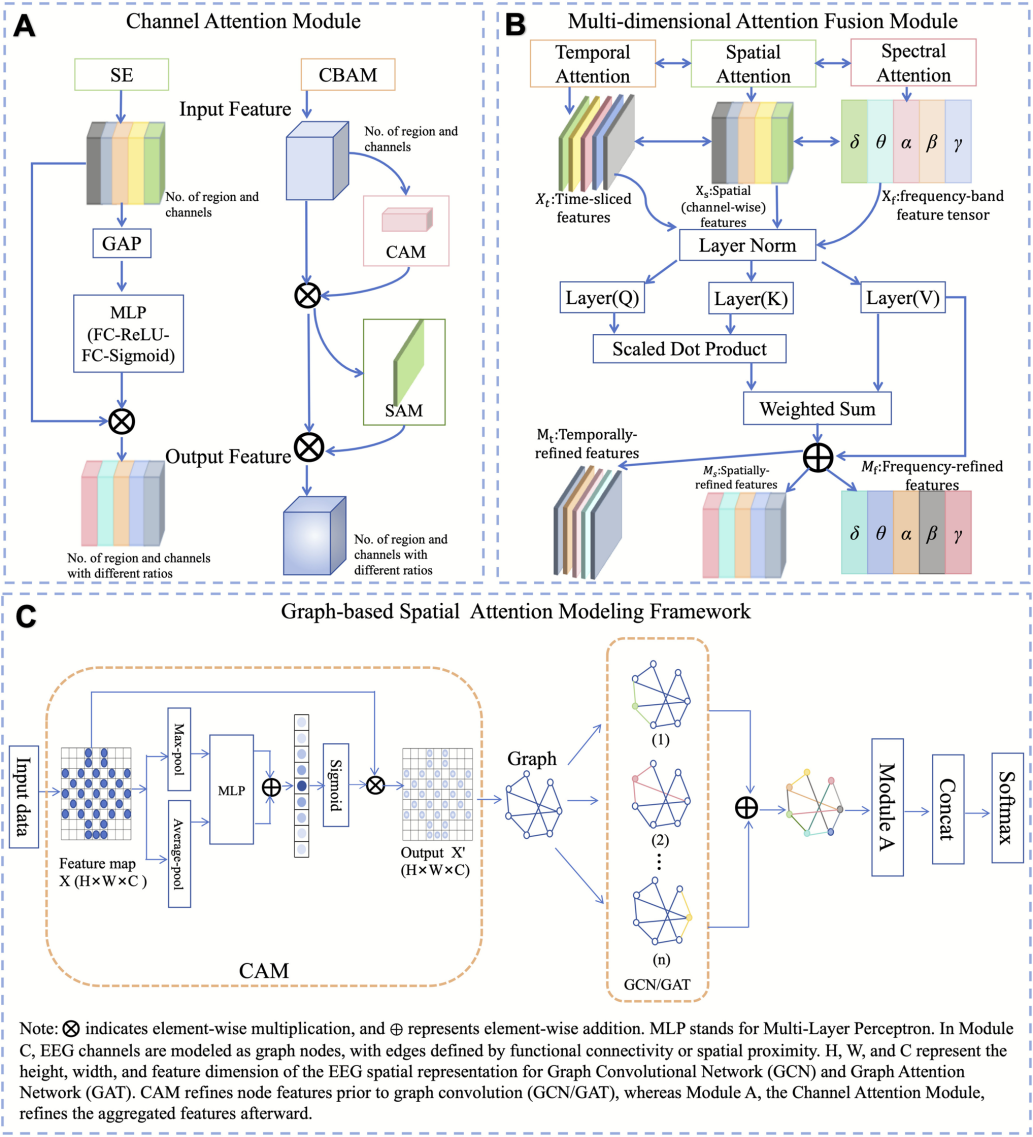
**A** Channel Attention Module; **B** Multi-head Attention Fusion Module; **C** Graph-based Spatial Modeling Framework for Spatial Attention in EEG-Based Emotion Recognition [28–31]

For instance, the weight computation of the SE module is formulated as in Eq. (1) (Fig. 3A, left).

1 $${s}_c=\sigma \left({W}_2\delta \left({W}_1{z}_c\right)\right)$$

Here, *z*_*c*_ denotes the global pooling feature of channel *c*, *δ* represents the ReLU function, and *σ*represents the Sigmoid activation. The process involves global feature compression, nonlinear mapping, and weight generation, ultimately producing an independent weight for each channel.

The CBAM module (Fig. 3A, right) further extends the approach by integrating global average and maximum features. The core computation is represented in Eq. (2).

2 $${M}_c(F)=\sigma \left( MLP\left( AvgPool(F)\right)+ MLP\left( MaxPool(F)\right)\right)$$

Here, *AvgPool* and *MaxPool* capture global average and maximum features respectively, which are then mapped through a shared multilayer perceptron and fused to obtain the final channel weight map *M*_*c*_(*F*). Compared with SE, CBAM incorporates multiple statistical descriptors in channel modeling and combines them with spatial attention, leading to more fine-grained feature selection.

### Multi-Head Attention Mechanism

The self-attention mechanism models global dependencies by computing a correlation matrix across channels or temporal segments. The multi-head mechanism further extends its representational capacity, enabling the model to capture diverse types of dependencies in parallel from multiple subspaces.

Self-attention leverages the interaction among queries (Q), keys (K), and values (V) to model global dependencies, with the computation formulated as in Eq. (3) [30].

3 $$Attention\left(Q,K,V\right)= softmax\left(\frac{Q{K}^T}{\sqrt{d^k}}\right)$$

Here, *d*^*k*^ denotes the dimensionality of the key vectors.

Multi-head attention computes attention distributions across multiple subspaces in parallel, thereby facilitating more comprehensive feature learning. The computation is expressed in Eq. (4) [30].

4 $$MultiHead\left(Q,K,V\right)= Concat\left( hea{d}_1,\dots, hea{d}_h\right){W}^O$$

Here, *h* denotes the number of heads and *W*^*O*^ represents the linear projection matrix for multi-head outputs.

In EEG-based emotion recognition, this mechanism has been extended to temporal, spatial, and spectral domains, allowing the capture of multidimensional dependencies. As illustrated in Fig. 3B, temporal attention emphasizes critical moments, spatial attention identifies key channels or brain regions, and spectral attention distinguishes the significance of rhythms such as α, β, and γ, thus achieving comprehensive modeling of EEG signals.

### Graph-Structured Modeling

EEG electrodes naturally form a non-Euclidean space, which is well suited for representation as a graph structure. Graph-based modeling explicitly characterizes nodes as channels and edges as inter-regional connections, with attention mechanisms incorporated to enhance the representation of dynamic dependencies. Representative approaches include GCN and GAT.

In GCN, node features are propagated through the adjacency matrix and the normalized Laplacian matrix, as expressed in Eq. (5) [32].

5 $${H}^{\left(l+1\right)}=\left.\sigma \left({\overset{\sim }{D}}^{-\frac{1}{2}}{\overset{\sim }{A}\overset{\sim }{D}}^{-\frac{1}{2}}\right){H}^{(l)}{W}^{(l)}\right)$$

Here, $\overset{\sim }{A}$ *= A +I*_*N*_ denotes the adjacency matrix with self-loops, *H*^(*l*)^ represents the node features at layer *l*, *W*^(*l*)^ is the learnable weight matrix, and *σ* denotes the nonlinear activation function. The formulation reflects a neighborhood aggregation process driven by a static adjacency matrix.

In comparison, GAT introduces adaptive weights based on attention coefficients. The node update process is expressed in Eq. (6) [33].

6 $$h{\prime}_i=\sigma \left({\sum}_{j\epsilon {N}_i}{\alpha}_{ij}W{h}_j\right),\kern1em {a}_{ij}=\frac{\mathit{\exp}\left( LeakyReLU\left({a}^T\left[\left.W{h}_i\right\Vert W{h}_j\right]\right)\right)}{\sum_{k\epsilon {N}_i}\mathit{\exp}\left( LeakyReLU\left({a}^T\left[\left.W{h}_i\right\Vert W{h}_j\right]\right)\right)}$$

In this expression, *α*_*ij*_ denotes the attention coefficient between node *i* and its neighbor *j* which is learned adaptively through a parameterized function. Unlike the fixed adjacency matrix in GCN, GAT allows connection weights to vary dynamically with the input, thereby aligning more closely with the time-varying nature of EEG functional connectivity.

In practical applications, graph-based methods are often integrated with the aforementioned attention mechanisms. Channel attention mechanisms are typically applied at the input stage to highlight brain regions strongly associated with emotional processing. Multi-head attention could be combined with graph modeling to dynamically weight node relationships and capture multidimensional dependencies. Serving as complementary modules, these mechanisms effectively enhance the capacity of models to learn complex spatial interactions.

## Advances in Graph-Based Spatial Attention Mechanisms

**Fig. 4:**
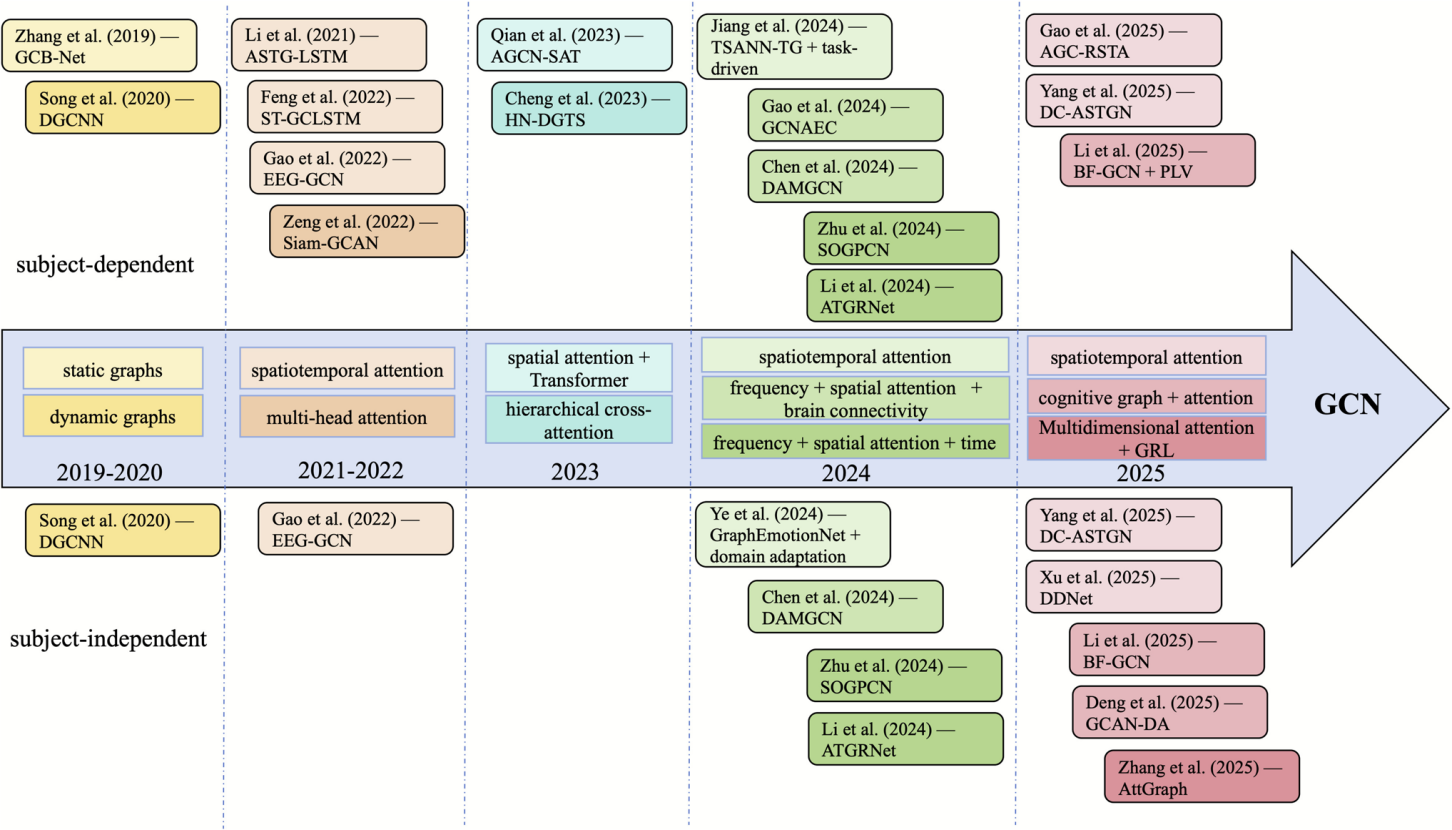
Temporal evolution of GCN-based spatial models

**Fig. 5:**
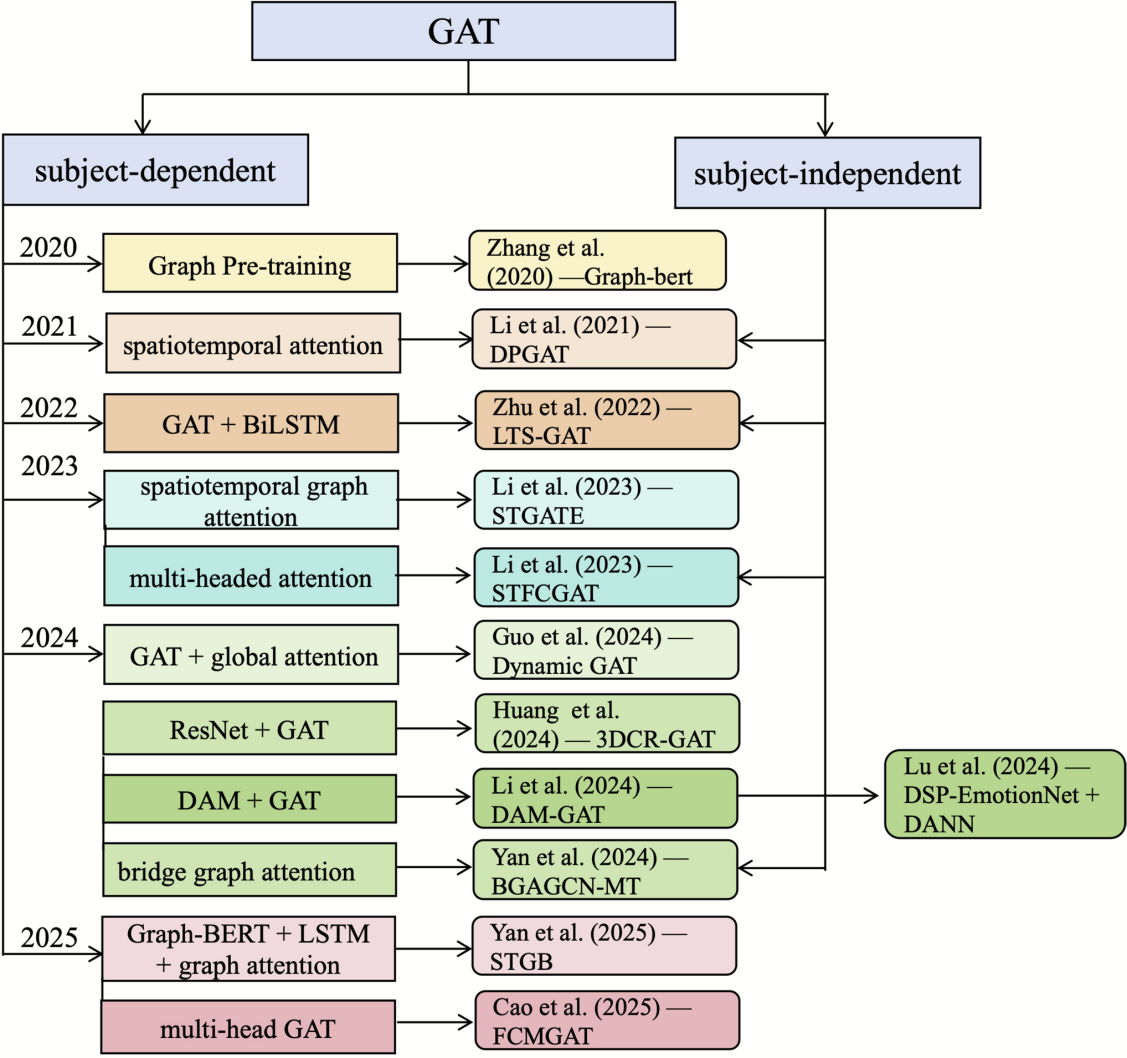
Temporal evolution of GAT-based spatial models

Topology modeling with GNN has become a primary strategy for addressing spatial dependencies in multi-channel EEG. As outlined in the preceding section, graph-based approaches such as GCN and GAT, when combined with attention mechanisms, provide powerful tools for explicitly modeling inter-channel interactions. The section examines the progression of these two technical paradigms across subject-dependent and subject-independent tasks, incorporating representative experimental insights and considerations of efficiency. It subsequently provides a comprehensive discussion on the distinctive advantages of aligning graph-based representations with neurophysiological consistency. Figures 4 and 5 summarize the temporal evolution of representative GCN- and GAT-based models from 2019 to 2025, while Tables 1 and 2 detail their architectures, innovations, and performance across datasets.

**Table 1:** Table of comparison of GCN-Based Methods with SD denotes subject-dependent and SI denotes subject-independent

Ref.	Authors/Year	Model	Design	Innovation	Limitation	Acc/Std
[34]	Zhang T, Wang X, Xu X, et al.(2019)	GCB-Net	Combines GCN with BLS, preserves multi-scale spatial info	• Preserves multi-scale features • Fits small-sample scenarios	• Relies on static graph • High computational cost	• SEED SD: 94.24 ± 6.70% • DREAMER SD ■ Valence: 86.99 ± 6.21% ■ Arousal: 89.32 ± 5.01% ■ Dominance: 89.20 ± 4.33%
[35]	Li X, Zheng W, Zong Y, et al.(2021)	ASTG-LSTM	Dynamic graph + spatiotemporal attention + LSTM	• Spatiotemporal collaboration • Dynamic graph construction	• Complex parameter tuning • High computational cost	• DEAP SD ■ Valence: 98.71 ± 0.80% ■ Arousal: 98.70 ± 0.64% • DREAMER SD ■ Valence: 96.27 ± 1.83% ■ Arousal: 96.44 ± 1.94% ■ Dominance: 85.02 ± 10.25%
[36]	Zeng H, Wu Q, Jin Y, et al. (2022)	Siam-GCAN	Siamese architecture + GCN + multi-head attention mechanism	• Efficient feature extraction • Discriminative feature learning	• Dependence on preprocessing • Sensitivity to small datasets	• SEED SD: 94.78 ± 5.97% • SEED-IV SD: 79.48 ± 15.14%
[37]	Feng L, Cheng C, Zhao M, et al.(2022)	ST-GCLSTM	PCC-based graph + SGCN + BiLSTM + attention mechanism	• Considers brain region topology • Spatiotemporal collaboration	• Uses static adjacency • High computational cost	• SEED SD: 96.72% • SEED-IV SD: 95.00% • DEAP SD: ■ Valence: 95.52% ■ Arousal: 95.04%
[13]	Qian B, Qian Z, Liang H, et al.(2023)	AGCN-SAT	Adaptive GCN + spatial attention + Transformer	• Flexible spatial modeling • Good subject-independent performance • Considers global features	• High computational cost • Attention interpretability is limited	• SEED SD: 92.76 ± 6.16%
[38]	Cheng C, Yu Z, Zhang Y, et al.(2023)	HN-DGTS	DGC + TSAR + H-CAF	• Spatiotemporal collaboration • Efficient feature fusion	• High computational cost • Strong data dependency	• SEED SD: 97.53% • SEED-IV SD: 98.97% • DEAP SD: 89.98%
[39]	Jiang C, Dai Y, Ding Y, et al.(2024)	TSANN-TG	task-specific adjacency matrices + spatiotemporal attention modeling	• Multi-scale signal optimization • Good generalization	• Weak in modeling individual differences • Model complexity is high	• DEAP SD ■ Valence: 63.74 ± 9.90% ■ Arousal: 65.93 ± 10.80% • FTEHD SD • HC Group: 92.88 ± 7.09% • Dep Group: 87.39 ± 9.32%
[40]	Gao P, Zheng X, Guo G, et al. (2024)	GCNAEC	CFC-driven graph construction + graph attention mechanism + graph processing with PPC	• Innovative graph construction	• Dependence on predefined networks	• DEAP SD ■ Valence: 83.24% ■ Arousal: 89.70%
[41]	Gao D, Zheng Q, Li P, et al. (2025)	AGC-RSTA	AGC + residual spatiotemporal attention mechanism to mitigate feature distortion	• Dynamic topology modeling • Good interpretability	• Parameter sensitivity	• SEED SD: 94.91 ± 5.84% • SEED-IV SD: 91.17 ± 9.38%
[42]	Song T, Zheng W, Song P, et al.(2020)	DGCNN	Proposes dynamic GCN by learning adjacency matrix adaptively	• Dynamic connectivity modeling • Enhanced spatial representation	• Focuses mainly on local modeling	• SEED SD: 90.40 ± 8.49% • SEED SI: 79.95 ± 9.02% • DREAMER SD ■ Valence: 86.23 ± 12.29% ■ Arousal: 84.54 ± 10.18% ■ Dominance: 85.02 ± 10.25%
[11]	Gao Y, Fu X, Ouyang T, et al.(2022)	EEG-GCN	Adaptive adjacency + spatiotemporal attention + multi-view fusion	• Spatiotemporal collaboration • Topology self-learning	• Relies on feature pre-extraction • Complex parameter tuning	• SEED SD: 85.65 ± 7.49% • SEED SI: 77.30 ± 8.21% • DEAP SD ■ Valence: 81.77 ± 5.58% ■ Arousal: 81.95 ± 7.71%
[43]	Chen W, Liao Y, Dai R, et al.(2024)	DAMGCN	Dual attention for channel connections and spectral weights	• Good interpretability • Multi-dimensional attention	• Low subject-independent performance • Prone to local optima	• SEED SD: 99.42 ± 0.24% • SEED SI: 73.21 ± 8.35% • SEED-IV SD: 96.86 ± 1.33% • SEED-IV SI: 68.22 ± 7.03% • DEAP SD ■ Valence: 96.96 ± 2.24% ■ Arousal: 97.17 ± 2.35% ■ Dominance: 97.50 ± 2.03% • DEAP SI: 64.61 ± 10.54%
[44]	Zhu X, Liu C, Zhao L, et al.(2024)	SOGPCN	Frequency-specific graph + pseudo-3D conv + attention + LSTM	• Optimized multi-band graph • Good subject-independent performance • Lightweight and efficient	• Relies on spectrum extraction • Performance varies across subjects	• SEED SD: 95.26 ± 3.52% • SEED SI: 94.22 ± 3.42%
[45]	Li C, Wang F, Zhao Z, et al.(2024)	ATGRNet	Chebyshev conv + hierarchical attention + TCN for parallel modeling	• Multidomain fusion • Good subject-independent performance • Efficient and robust time modeling • Good interpretability	• Sensitive to data quality • Relies on spectrum extraction	• SEED SD: 92.59 ± 8.73% • SEED SI: 87.55 ± 5.25% • DEAP SD ■ Valence: 78.22 ± 18.33% ■ Arousal: 76.46 ± 19.48% • DEAP SI ■ Valence: 68.65 ± 9.09% ■ Arousal: 68.75 ± 7.85% • FACED SD: 87.97 ± 13.99% • FACED SI: 75.76 ± 5.58%
[46]	Yang X, Zhu Z, Jiang G, et al. (2025)	DC-ASTGN	DCNN + ASTGCN + spatiotemporal attention	• Multidomain fusion • Stable model performance	• Limited subject-independent generalization • High computational cost	• SEED SD: 87.65 ± 7.54% • SEED SI: 80.65 ± 8.46% • DEAP SD ■ Valence: 93.36 ± 1.92% ■ Arousal: 94.42 ± 1.78% • DEAP SI ■ Valence: 90.43 ± 1.48% ■ Arousal: 91.15 ± 2.05%
[47]	Xu B, Zhang X, Zhang X, et al. (2025)	DDNet	DAM + DGC + adapter	• Good spatial feature extraction • Strong adaptability	• Performance fluctuates across individuals	• SEED SI: 92.63% • SEED-IV SI: 85.03%
[48]	Ye W, Wang J, Chen L, et al. (2024)	GraphEmotionNet	Adaptive graph learning + spatio-temporal attention + DANN	• Spatiotemporal collaboration • Strong domain adaptation • High stability	• High computational cost • Feature dependence	• SEED SI: 86.43% • SEED-IV SI: 63.07% • MDD SI ■ closed-eye: 62.25% ■ open-eye: 63.64%
[49]	Li C, Tang T, Pan Y, et al. (2025)	BF-GCN	Combines PLV-based cognitive graph and self-learned graph + attention	• Good interpretability • Multi-graph fusion • Strong subject-independent robustness	• Cognitive graphs may be unstable • Lacks cross-dataset validation	• SEED SD: 97.44 ± 3.89% • SEED SI: 92.72 ± 3.90% • SEED-IV SD: 89.55 ± 10.95% • SEED-IV SI: 82.03 ± 8.42%
[50]	Deng X, Li Y, Ai L, et al. (2025)	GCAN-DA	Multi-branch GCN capturing local/global brain connectivity + attention mechanism + GRL	• Good subject-independent performance • Multi-scale feature fusion	• Large computational overhead • Requires broader validation	• SEED SI: 87.33%
[51]	Zhang S, Chu C, Zhang X, et al. (2025)	AttGraph	Multidimensional attention convolution + global attention + GRL	• Good subject-independent performance • Dynamic feature selection	• Limited interpretability • Single-subject age range	• SEED SI: 85.22 ± 4.90% • SEED-IV SI: 78.36 ± 9.61%

The reported accuracy (Acc) and standard deviation (Std) values are highly dependent on preprocessing pipelines and validation protocols, as referred to in Section Variability in Validation Protocols 5.1.2 and Table 3. Therefore, these results should not be interpreted as absolute rankings of model superiority

**Table 2:** Table of the comparison of Graph-Attention-Based Methods with SD denotes subject-dependent, and SI denotes subject-independent

Ref.	Authors/Year	Model	Design	Innovation	Limitation	Acc/Std
[52]	Zhang J, Zhang H, Xia C, et al.(2020)	Graph-BERT	sampled linkless subgraphs + attention for flexible graph modeling	• Scalable to large graphs • Alleviates over-smoothing	• No temporal modeling • EEG applicability unverified	–
[53]	Yan J, Du C, Li N, et al. (2025)	STGB	Graph-BERT + LSTM + graph attention	• Captures dynamic spatiotemporal EEG traits • Strong subject-independent robustness	• LSTM struggles with long-term dependency	• SEED SD: 89.39% • DEAP SD ■ Valence: 81.64% ■ Arousal: 80.32%
[54]	Cao M, Dong Y, Chen D, et al. (2025)	FCMGAT	Fuzzy adjacency matrix + GCN + multi-head GAT	• Good adaptability and stability of graph structure • Strong information capturing ability	• Relatively high complexity • Weak temporal modeling	• SEED SD: 97.41 ± 2.38% • SEED-IV SD: 92.08 ± 3.69%
[55]	Li J, Pan W, Huang H, et al.(2023)	STGATE	Transformer + spatiotemporal graph attention to construct dynamic structure	• Supports long-term dependencies • Good subject-independent performance	• Limited by small sample size • High computational cost	• SEED SD: 90.37 ± 6.18% • SEED-IV SD: 76.43 ± 5.01% • DREAMER SD ■ Valence: 77.44 ± 8.40% ■ Arousal: 75.26 ± 9.71%
[56]	Guo Y, Tang C, Wu H, et al. (2024)	Dynamic GAT	Dynamic directed graph + GAT + global attention	• Individualized modeling • High robustness	• Dependent on electrode physical locations	• SEED SD: 94.60 ± 4.98%
[57]	Huang Y, Liu T, Wu Q. et al. (2024)	3DCR-GAT	3D convolution + ResNet + GAT	• Innovative preprocessing • Joint modeling of spatio-temporal and channel relations	• Unstable neutral emotion recognition • High computational cost	• SEED SD: 92.00 ± 4.66%
[31]	Li C, Pun SH, Li JW, et al. (2024)	DAM-GAT	Dual-branch attention module (DAM) + GAT	• Captures functional correlations in EEG • Joint modeling of channel and frequency	• Complex model architecture	• SEED SD: 94.63%
[58]	Li X, Li J, Zhang Y, et al. (2021)	DPGAT	Dual-branch GAT + CNN + BiLSTM	• Spatiotemporal collaboration • Good subject-independent generalization • Good interpretability	• Lacks prior knowledge integration	• SEED SD: 95.76 ± 5.77% • SEED SI: 87.07 ± 6.31%
[14]	Zhu Y, Gan K, Yin Z(2022)	LTS-GAT	GAT + BiLSTM + domain adversarial learning for robustness	• Spatiotemporal collaboration • Good individual generalization	• Stability needs improvement • EEG-only	• DEAP SD ■ Valence: 66.74% ■ Arousal: 63.39% • DEAP SI ■ Valence: 66.17% ■ Arousal: 61.02% • HCI SD ■ Valence: 57.71% ■ Arousal: 65.76% • HCI SI ■ Valence: 54.03% ■ Arousal: 56.11%
[59]	Lu W, Zhang X, Xia L, et al.(2024)	DSP-EmotionNet	Dual-branch SATFEM + GAT + DANN + ResNet	• Strong domain generalization • Comprehensive feature extraction	• High computational cost • Lacks real-time performance	• SEED SI: 82.5 ± 7.6% • SEED-IV SI: 65.9 ± 7.8%
[60]	Yan H, Guo K, Xing X, et al. (2024)	BGAGCN-MT	deep graph convolution path (DGCP) + bridge graph attention (BGAT) + MT	• Mitigates over-smoothing • Multi-scale feature fusion	• High computational cost • Dependence on brain-region priors	• SEED SD: 96.82 ± 4.85% • SEED SI: 89.66 ± 4.72% • SEED-IV SD: 82.86 ± 9.31% • SEED-IV SI: 75.78 ± 8.17% • DREAMER SD ■ Valence: 93.92 ± 8.58% ■ Arousal: 94.60 ± 7.29% ■ Dominance: 94.75 ± 6.84%
[61]	Li Z, Zhang G, Wang L, et al.(2023)	STFCGAT	single-channel DE + cross-channel FC + CGAT+ and multi-headed attention	• Multidomain feature fusion • Strong generalization	• Weak temporal modeling • High computational cost	• SEED SD: 99.11 ± 0.83% • SEED SI: 94.83 ± 3.41% • DEAP SD ■ Valence: 95.70 ± 3.36% ■ Arousal: 95.04 ± 3.02% • DEAP SI ■ Valence: 91.19 ± 4.24% ■ Arousal: 92.03 ± 4.57%

The reported accuracy (Acc) and standard deviation (Std) values are highly dependent on preprocessing pipelines and validation protocols, as referred to in Variability in Validation Protocols Section 5.1.2 and Table 3. Therefore, these results should not be interpreted as absolute rankings of model superiority

### Graph Convolutional Network-Based Spatial Modeling

#### Subject-Dependent Optimization of GCN Modeling

In subject-dependent tasks, research on GCN has focused on the evolution from static graph structures to dynamic graphs capable of capturing emotion-related temporal variations, along with the integration of multidimensional attention mechanisms. In previous work, Defferrard et al. [62] first introduced spectral graph convolution into EEG analysis, establishing the theoretical foundation. However, the reliance on fixed adjacency matrices limited its ability to characterize dynamic features of emotional processing across brain regions. To address the limitation, Zhang et al. [34] integrated GCN with broad learning systems (BLS), which preliminarily enhanced feature fusion capabilities but still failed to overcome the constraints of static adjacency matrices.

As research has progressed, scholars have increasingly introduced attention mechanisms and dynamic graphs into EEG modeling to better capture spatiotemporal dependencies. Li et al. [35] proposed the attention-based spatiotemporal graphic long short-term memory (ASTG-LSTM) model to meet the increasing demands for modeling spatiotemporal dependencies. Zeng et al. [36] developed Siam-GCAN, which enhanced classification discriminability through a Siamese network structure. Feng et al. [37] integrated BiLSTM to extract deep temporal features, while Qian et al. [13] fused spatial attention with Transformer to achieve coordinated local and global modeling. Building upon these advances, Cheng et al. [38] introduced the HN-DGTS architecture, which combined temporal self-attention with hierarchical cross-attention to enable more fine-grained multidimensional feature integration.

In terms of graph structure optimization, Jiang et al. [39] proposed TSANN-TG, which explored task-driven adjacency matrix generation. Gao et al. [40] leveraged cross-frequency coupling (CFC) to construct functional connectivity adjacency matrices, incorporating graph attention mechanisms to capture global dependencies. More recently, another Gao et al. [41] proposed an adaptive GCN with residual attention (AGC-RSTA) to extract spatio-temporal discriminative features, further enhancing model stability.

Taken together, subject-dependent studies suggest that performance improvements are primarily driven by dynamic graph modeling and the integration of multidimensional attention mechanisms. These approaches effectively capture fine-grained spatiotemporal patterns at the individual level, thereby substantially improving the accuracy of emotion recognition. Meanwhile, task-driven and cross-frequency-based graph structure optimization shows considerable potential for further enhancing the robustness and generalizability of models.

#### GCN Modeling for Subject-Independent Generalization

In the more challenging subject-independent setting, the evolution of GCNs has shifted from purely data-driven approaches toward a deeper integration of cognitive priors, multidimensional feature fusion, and domain adaptation. Early attempts, such as DGCNN [42], adopted adaptive adjacency matrix updates for dynamic modeling, but exhibited limited generalization capacity.

To enhance performance, researchers have explored multiple directions. Building on DGCNN, Cao et al. [11] incorporated spatiotemporal attention to quantify dynamic variations in inter-regional connectivity under different emotional states. Chen et al. [43] proposed DAMGCN, which leverages Transformer modules to separately model regional connectivity and frequency-band weights. Zhu et al. [44] constructed independent self-organized graphs for multiple frequency bands with pseudo-3D convolution for feature extraction. The lightweight design delivered efficiency advantages while sustaining strong performance, indicating dual optimization under subject-independent settings. Li et al. [45] applied hierarchical attention to weight both channels and frequency bands, replacing LSTM with temporal convolutional networks (TCN) for efficient temporal modeling. Yang et al. [46] integrated deep convolutional networks with adaptive graph structures to capture spectral, spatial, and topological features simultaneously. Xu et al. [47] proposed DDNet, which employed multi-head attention for spatio-spectral modeling and introduced adapter modules to enhance subject-independent adaptability.

Other works have introduced cognitive priors and domain adaptation to mitigate inter-subject variability. Ye et al. [48] presented GraphEmotionNet, integrating spatio-temporal attention with domain adaptation on dynamic graphs, reducing inter-subject distribution gaps. Li et al. [49] introduced BF-GCN, which fused PLV-based functional connectivity priors with data-driven adaptive graphs, providing stable physiological structural constraints. Deng et al. [50] combined multi-branch fusion with domain adversarial networks to enhance both stability and structural learning. Similarly, Zhang et al. [51] applied gradient reversal layers (GRLs) to explicitly mitigate distribution shifts, thereby improving discriminative ability and generalization.

Overall, advancements in subject-independent tasks rely heavily on integrating cognitive priors, multidimensional feature fusion, and domain adaptation. GCN-based models primarily encode structured spatial priors and inter-channel dependencies, thereby offering topology-aware feature representations that are more robust to noise and local variability [11]. Conversely, domain adaptation modules, such as DANN or GRL, explicitly address inter-subject variability by aligning feature distributions between source and target subjects at the representation level. When these components are integrated, they function at complementary levels [63]. Spatial modeling based on graphs establishes a physiologically grounded and structured feature space. Domain adaptation operates within this space to reduce inter-subject distribution shifts. GNNs recognise the encoded spatial patterns, while domain adaptation ensures their consistency across individuals [50, 51]. Consequently, this configuration provides a rigorous methodology for developing more robust, subject-independent frameworks for EEG-based emotion recognition.

#### Experimental Insights on Preprocessing, Performance, and Efficiency

Recent studies indicate that increased architectural complexity frequently yields diminishing returns unless complemented by meticulous preprocessing. The GCAN-DA model integrates multi-branch feature fusion, domain adversarial networks, and attention mechanisms [50]. During preprocessing, a band-pass filter spanning 0 to 75 Hz with an ICA module for noise reduction, followed by the computation of PLV to evaluate inter-regional synchronization. Furthermore, linear dynamical system (LDS) was implemented to remove extraneous components and is shown in Table 3. In subject-independent tasks utilising the SEED dataset, the GCAN-DA model demonstrated superior performance in comparison to the AttGraph model developed by Zhang [51]. Although AttGraph also employed a GRL mechanism, its filtering and segmentation strategies were less meticulous, thereby constraining the shared feature representations obtained under subject-independent conditions. Consequently, AttGraph serves as a prominent failure example in subject-independent settings within this experimental framework, highlighting that insufficient preprocessing rigor can substantially impair subject-independent generalization, even when domain adaptation modules are incorporated.

**Table 3:** Preprocessing and performance of graph-based spatial attention models (SD and SI)

Ref.	Authors/Year	Model	Preprocessing	Dataset & Accuracy
[41]	Gao D, Zheng Q, Li P, et al. (2025)	AGC-RSTA	• Bandpass: δ, θ, α, β, γ • Features: DE per band • Windowing after DE: sliding windows, fixed size, step 1–3 s	• SEED SD: 94.91 ± 5.84% • SEED-IV SD: 91.17 ± 9.38%
[46]	Yang X, Zhu Z, Jiang G, et al.(2025)	DC-ASTGN	• Windowing: 1 s segments with 0.5 s time-steps • Bandpass: δ, θ, α, β, γ • Features: 5-band DE • Feature construction: 2D Electrode Mapping	• SEED SD: 87.65 ± 7.54% • SEED SI: 80.65 ± 8.46% • DEAP SD ■ Valence: 93.36 ± 1.92% ■ Arousal: 94.42 ± 1.78% • DEAP SI ■ Valence: 90.43 ± 1.48% ■ Arousal: 91.15 ± 2.05%
[47]	Xu B, Zhang X, Zhang X, et al. (2025)	DDNet	• Windowing: 1 s per segment • Features: 5-band DE	• SEED SI: 92.63% • SEED-IV SI: 85.03%
[49]	Li C, Tang T, Pan Y, et al. (2025)	BF-GCN	• Windowing: 1 s per segment • Bandpass: δ, θ, α, β, γ • Features: 5-band DE + PLV	• SEED SD: 97.44 ± 3.89% • SEED SI: 92.72 ± 3.90% • SEED-IV SD: 89.55 ± 10.95% • SEED-IV SI: 82.03 ± 8.42%
[50]	Deng X, Li Y, Ai L,et al. (2025)	GCAN-DA	• Downsample: 200 Hz • Bandpass: 0–75 Hz + ICA • Windowing: 1 s per segment • Features: 5-band (δ,θ,α,β,γ) DE + PLV (phase-locking value for inter-region connectivity) • Filtering: Linear Dynamic System (LDS) to remove irrelevant components	• SEED SI: 87.33 ± 5.32%
[51]	Zhang S, Chu C, Zhang X, et al. (2025)	AttGraph	• Downsample: 200 Hz • Bandpass: 0.3–50 Hz, noise removal • Artifact rejection: manual removal of EMG/EOG contaminated segments • Windowing: 1 s per segment • Features: 5-band DE, PSD, etc.	• SEED SD: 97.45 ± 2.20% • SEED SI ■ DE: 85.22 ± 4.90% ■ PSD: 80.99 ± 5.21% • SEED-IV SD: 93.92 ± 2.78% • SEED-IV SI ■ DE: 78.36 ± 9.61% ■ PSD: 62.97 ± 9.42%
[53]	Yan J, Du C, Li N, et al. (2025)	STGB	• Windowing: SEED 3 s, DEAP 4 s • Bandpass: δ, θ, α, β, γ • Features: DE, PSD, DASM, RASM, DCAU	• SEED SD: 89.39% • DEAP SD ■ Valence: 81.64% ■ Arousal: 80.32%
[54]	Cao M, Dong Y, Chen D, et al. (2025)	FCMGAT	• Windowing: 0.4 s per window, 0.2 points step • Butterworth filter: remove δ band (0.5–4 Hz) • Features: DE and PSD • Also retain raw signal as model input • SEED SD: 94.91 ± 5.84%	• SEED SD ■ DE: 97.412% ■ PSD: 88.825% • SEED-IV SD ■ DE: 92.086% ■ PSD: 74.122%

The trade-off between computational efficiency and model scalability remains a pivotal challenge for the deployment of BCI in real-world applications. Although GCNs typically provide faster inference than attention-intensive networks, due to the potential use of static or precomputed adjacency matrices, the recent shift towards dynamic graphs markedly increases computational requirements [46, 50]. Further, the GCAN-DA model employs multi-branch fusion and sophisticated artifact removal techniques to attain high accuracy [50]. Nevertheless, these components introduce considerable latency, which may be prohibitive for real-time closed-loop systems. Conversely, lightweight architectures such as pseudo-3D convolution demonstrate that structural pruning can preserve scalability without significantly compromising accuracy [44]. Future development of GCNs must therefore strike a balance between the comprehensive feature representation afforded by dynamic topologies and the low-latency requirements characteristic of wearable devices.

### Graph Attention Mechanism-Based Spatial Modeling

#### Subject-Dependent Optimization of GAT Modeling

Unlike GCNs that rely on fixed weights, GATs assign dynamic weights to neighboring nodes through self-attention, enabling finer-grained capture of inter-node importance differences [33]. This adaptive weighting enables GATs to capture subject-specific heterogeneity and sparse EEG connectivity.

In subject-dependent tasks, researchers have attempted to integrate GATs with multidimensional features to exploit dynamic weighting capabilities. Zhang et al. [52] proposed Graph-BERT, which employed linkless subgraph sampling to alleviate over-smoothing and improve the efficiency of large-scale graph processing. In addition to subgraph sampling, common strategies to mitigate over-smoothing in dense EEG graphs include residual or skip connections [64, 65], edge dropout [66, 67], and shallow attention stacking [68, 69], which assist in maintaining discriminative node representations within deep GAT architectures. In the Graph-BERT framework, Yan et al. [53] developed the STGB, combining Graph-BERT with LSTM and graph attention for joint spatio-temporal modeling. Cao et al. [54] presented FCMGAT, which incorporates fuzzy logic to smooth graph structures, thereby enhancing model adaptability and training stability. Li et al. [55] integrated Transformer encoders with spatiotemporal graph attention to support dynamic graph construction and capture cross-regional dependencies. Guo et al. [56] employed GAT with a global attention module to jointly capture more discriminative emotional features. Similarly, Huang et al. [57] combined 3D convolution with residual structures to capture local spatiotemporal features, and further proposed the Longest Trial Baseline Alignment (LTBA) strategy to improve robustness. Beyond spatiotemporal modeling, the frequency domain has also emerged as a promising direction. Li et al. [31] designed a dual-branch attention module that dynamically optimizes channel and frequency features, then combined GAT to capture inter-regional interactions, achieving over 94% accuracy on the SEED dataset.

Table 2 indicates that GAT models fused with spatio-temporal or spectral-spatial attention achieved outstanding results in subject-dependent tasks, confirming that dynamic weight allocation could capture individual-specific emotional patterns more precisely, especially when optimized jointly with multidomain features.

#### GAT Modeling for Subject-Independent Generalization

In subject-independent tasks, the substantial structural variability across individuals poses significant challenges, making the improvement of GAT generalization and robustness a central research focus. Li et al. [58] introduced DPGAT with dual branches to model spatial dependencies and temporal sequences, providing initial evidence of GAT potential in subject-independent scenarios. Zhu et al. [14] further enhanced robustness by integrating GAT with BiLSTM and a dynamic domain discriminator. Lu et al. [59] proposed DSP-EmotionNet, which integrated residual network (ResNet), GAT, and domain-adversarial learning, improving stability and generalization simultaneously. Yan et al. [60] introduced a bridging graph attention convolutional network to mitigate the over-smoothing problem in deep graph models. Multidimensional feature fusion has also proven effective for enhancing subject-independent performance. Li et al. [61] proposed the STFCGAT model, an advanced framework integrating single-channel differential entropy (DE) and cross-channel functional connectivity (FC) features with CGAT and multi-head graph attention, achieving 94.83% accuracy on SEED.

Overall, GAT models in subject-independent settings mainly rely on multi-branch architectures, domain adaptation techniques, and multidomain feature fusion to improve their generalization. These strategies help address individual differences and highlight GAT’s advantages in dynamic modeling and cross-domain adaptation. Functionally, GATs assign weights to inter-channel connections dynamically, enabling flexible subject-specific and context-dependent spatial modeling [58]. Additionally, domain adaptation modules enhance robustness by aligning learned representations across subjects [59]. This design supports the suitability of GAT-based frameworks for subject-independent EEG modeling in heterogeneous conditions.

#### Experimental Insights on Feature Selection, Windowing, and Efficiency

Experimental comparisons indicate that model effectiveness is significantly influenced by temporal parameters within experimental settings. For example, AttGraph model by Zhang [51], previously noted for preprocessing constraints in subject-independent tasks, employed a 1-second windowing approach advantageous in subject-dependent scenarios. This setup enhanced performance on the SEED-IV dataset compared to FCMGAT model by Cao [54], as shown in Table 3. Although FCMGAT utilized multi-head graph attention mechanisms to capture global channel dependencies, it used shorter 0.4-second windows and lacked explicit temporal modeling. Such limitations are especially evident in demanding or variable conditions, where short window segmentation and absent explicit temporal modeling reduce robustness and transferability. These findings suggest that combining windowing strategies often yields greater benefits than relying solely on spatial dependency modeling.

Furthermore, computational scalability remains a concern for GATs versus GCNs. Pairwise attention coefficient calculations scale quadratically with node count, creating bottlenecks in high-density EEG applications. While GATs improve interpretability by highlighting important connections, the cost of multi-head attention may hinder deployment on resource-limited edge devices. State-of-the-art models like STFCGAT achieve high accuracy through complex multidomain fusion, but this often prolongs inference times [61]. Future models migght prioritize computational efficiency compatible with portable hardware to facilitate translating BCI technology into clinical use. Strategies such as linear attention or sparse graph learning could maintain the adaptive advantages while reducing computational load.

### Graph-Structured Modeling and Neurophysiological Consistency

Graph-based spatial modeling aligns naturally with the distribution of EEG electrodes and the functional connectivity of brain regions, as nodes and edges explicitly represent these relationships. This approach not only enhances the precision of spatial feature extraction but also improves interpretability. The correspondence between graph structures and neural functions allows model decisions to be cross-validated against neuroscientific theories.

Several studies have explored this integration. Gao et al. [40] combined GCN with graph attention to capture inter-regional dependencies and global feature distributions. Li et al. [43] constructed functional brain connectivity graphs using PLV and incorporated them into graph modeling, proposing a method that integrates neural priors with data-driven learning. Gao et al. [41] further demonstrated through visualization that their model assigned greater weights to prefrontal and parietal regions during emotion recognition, a finding consistent with established knowledge of emotion-related brain regions. These results suggest that graph-based modeling not only improves recognition performance but also provides distinct advantages in interpretability.

### Summary of Graph-Based Spatial Modeling

In summary, advances in GCN and GAT architectures have transformed EEG emotion recognition, shifting from single-channel analysis to network-level topological modeling. GCNs often utilize structured priors to enhance stability and encode cognitive constraints, while GATs use attention mechanisms to capture heterogeneity and local dependencies. Despite these differences, both follow similar developmental paths. Figures 4 and 5 show the literature progressing from static to adaptive adjacency modeling for better representation of time-varying functional connectivity. Additionally, there is a move from single-domain to multispectral integration across temporal, spatial, and spectral features to improve feature complementarity and discrimination. More studies now adopt hybrid strategies combining neural priors with domain adaptation techniques to enhance generalization across subjects and datasets. Graph-based modeling also aligns computational representations more closely with neuroscientific interpretations, reducing dependence on opaque black-box explanations. However, key challenges remain, including limited robustness in subject-independent scenarios, high computational costs, and partial validation of physiological relevance.

## Experimental Factors and Reproducibility in Graph-Based EEG Modeling

The previous sections have examined the development of spatial attention mechanisms and demonstrated that the experimental setup, technical variations in datasets, preprocessing techniques, and validation procedures significantly impact reported performance metrics. These factors are often as influential as, or even more influential than, architectural design considerations. Although the architecture of a model theoretically defines the maximum achievable performance, the actual realization of this potential in empirical research is heavily dependent on experimental factors. This section offers a systematic analysis of these elements and introduces a standardized checklist aimed at enhancing reproducibility and promoting equitable comparisons.

### Implicit Influence of Experimental Factors

#### Dataset Heterogeneity

Current research relies heavily on several widely used public datasets, yet fundamental design differences of these datasets create inherent dependencies in reported performance. For example, the DEAP dataset [20] employs dimensional emotion labels based on valence and arousal, with relatively long trial durations, which favors models capable of capturing long-term temporal dynamics and performing regression analysis. By contrast, the SEED dataset and its derivatives [70, 71] adopt discrete emotion labels and higher channel density, making them more suitable for evaluating the capacity of graph-based models to capture complex spatial topologies. Table 3 shows that the models exhibit variable performance across datasets, despite utilising similar architectural frameworks. This highlights the substantial dataset dependence of the reported outcomes. Such variability suggests that enhancements observed in a single dataset may not be consistently replicable across other experimental settings and claims of generalizability should therefore be regarded with caution.

#### Variability in Preprocessing Pipelines

Even minor differences in preprocessing procedures often produce amplified effects that significantly influence final performance. Critical steps include sampling rate adjustment and baseline correction, temporal window segmentation, frequency band selection, artifact removal methods such as manual rejection versus automatic independent component analysis, and feature construction strategies such as DE, PSD, or their combination. Each choice involves inherent trade-offs, with short windows increasing the number of training samples but weakening global emotional dynamics, whereas long windows preserve richer temporal information at the cost of reduced sample size; frequency filtering may inadvertently remove important emotional information; and the strictness of artifact removal directly affects subject-independent generalization. Table 3 shows substantial performance variations across works employing similar model architectures and datasets but different preprocessing pipelines. In the absence of standardized protocols, the same model often exhibits large performance variations across different studies, making it difficult to assess the true contribution of reported incremental improvements.

#### Variability in Validation Protocols

The choice of validation protocol has a critical impact on performance evaluation. Subject-dependent experiments typically achieve higher accuracy, yet the results primarily reflect individual-specific characteristics and lack cross-population generalization. In contrast, subject-independent experiments provide a more rigorous assessment of robustness in practical applications, although such experiments are often accompanied by a substantial decline in performance. Table 3 shows that numerous recent graph-based EEG models exhibit differences between subject-dependent and subject-independent configurations across datasets. This highlights the sensitivity of the reported outcomes to the design of validation protocols. However, the existing literature lacks consistency and transparency on the protocols. It might lead to unequal performance comparisons across studies and amplify ambiguity in the interpretation of reported progress.

Overall, dataset heterogeneity, variability in preprocessing pipelines, and inconsistency in validation protocols jointly determine the comparability and reproducibility of reported performance. These implicit factors frequently hinder the verification of claimed improvements across studies. Future research must therefore place greater emphasis on transparency and standardization of experimental design. Clear documentation of data partitioning, unified preprocessing procedures, and rigorous validation protocols are essential for establishing reliable baselines and advancing EEG-based emotion recognition from dataset dependency toward genuine cross-scenario generalization.

### Synthesis and Recommendations for Reproducible Graph-Based EEG Modeling

**Fig. 6:**
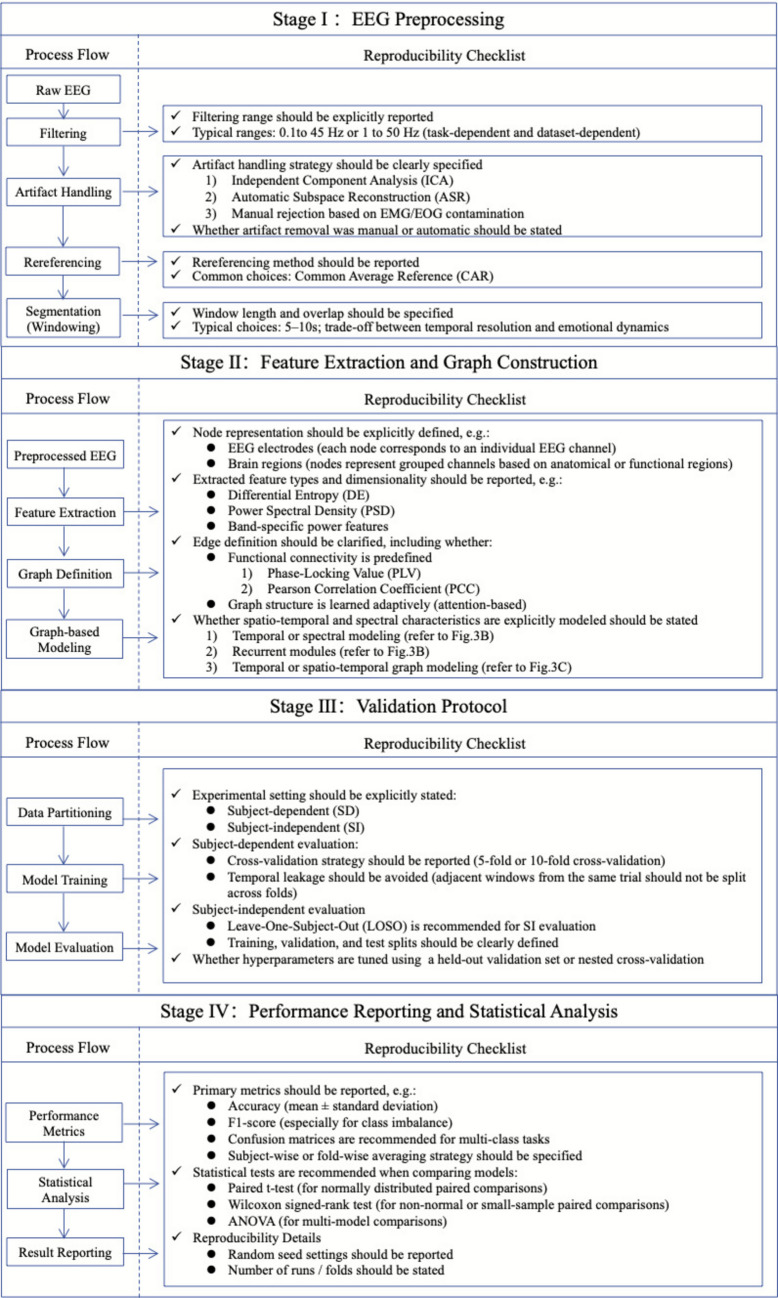
A proposed framework or checklist for Graph-Based EEG for emotion recognition

Figure 6 summarizes the reproducibility of the experimental framework for graph-based EEG emotion recognition. The framework and protocols aim to address variability in preprocessing pipelines and validation processes. The framework or checklist organizes the main experimental stages, including preprocessing, feature and graph construction, validation protocols, and performance reporting, with corresponding checklists to facilitate transparent reporting and equitable comparison across studies. The proposed framework has the potential to promote transparency in interpretation and adaptation across specific datasets, tasks, and experimental conditions.

## Future Directions and Research Trends

Although spatial attention-based EEG emotion recognition has advanced considerably, existing approaches still face challenges in model efficiency, neurophysiological interpretability, multimodal generalization, and practical translation to neuroscience and clinical applications. Future research should focus on the following directions.

## Lightweight Modeling and Resource Adaptation

Most spatial modeling approaches achieve impressive accuracy but rely on complex architectures with redundant parameters and high computational cost, thereby limiting the applicability of such models to wearable or edge devices [11, 35]. Zhu et al. [44] introduced a pseudo-3D convolution framework to reduce computational demand, though the subject-independent performance remained unstable. Future work may combine lightweight architectures with dynamic structure pruning to balance expressiveness and efficiency. Conditional computation and attention routing could further reduce inference costs while preserving essential spatial features, thereby facilitating real-time and low-power applications.

### Multimodal and Subject-Independent Integration

Achieving robust and generalizable EEG emotion recognition requires simultaneously enhancing spatial representation and mitigating individual variability. EEG signals, while temporally precise, suffer from low spatial resolution and susceptibility to noise, limiting their reliability in complex emotional states [23]. To address these limitations, Jia et al. [72] proposed MDNet, a multimodal framework that integrates EEG with fNIRS and eye-tracking via multi-head attention, thereby improving spatial characterization and cross-modal complementarity. In parallel, Li et al. [45] developed a top-k spectral graph network with hierarchical attention to jointly model channels and frequency bands, demonstrating improved cross-dataset generalization. Future work should pursue unified multimodal graph frameworks that combine adaptive topology learning and domain-invariant representations, enabling joint exploitation of neural and peripheral signals for more stable, transferable, and ecologically valid emotion recognition systems. Strengthening such integrative and neurophysiologically informed modeling not only enhances algorithmic robustness but also provides a foundation for translational applications in affective neuroscience and clinical practice.

### Neurophysiological Alignment

Despite significant accuracy improvements, current spatial models often fail to align with actual neural mechanisms. Graph structures are usually defined by electrode layouts or heuristic rules [73], rather than grounded in functional connectivity, which weakens both physiological interpretability and inter-subject consistency [46]. Li et al. incorporated PLV priors to introduce biological constraints, yet stability and generalizability issues persist [49].

In the architectural design, neurophysiological alignment should be explicitly evaluated. One feasible approach is to compare learned graph structures or attention-derived edge weights with established functional connectivity measures (e.g., PLV or coherence) using correlation or similarity analyses, thereby assessing the physiological plausibility of learned spatial dependencies [74, 75]. Complementary visualization of region-level activation, hemispheric asymmetry, and emotion-related connectivity patterns, particularly their consistency across subjects, can further verify whether the model captures biologically meaningful mechanisms rather than incidental statistical correlations [76, 77]. Integrating these strategies could enhance both the biological validity and scientific credibility of spatial attention-based EEG emotion recognition.

### Clinical and Neuroscientific Applications

EEG-based emotion recognition has growing potential in neuroscience and clinical medicine. Existing studies have applied EEG-based emotion decoding to the detection of anxiety, depression and post-traumatic stress, revealing associations between neural oscillatory signatures and affective dysregulation [3]. Neurofeedback and affective brain-computer interfaces have been used to modulate mood states and improve therapeutic outcomes in patient populations [4]. Combining EEG emotion analysis with neuroimaging further facilitates mapping of emotional circuits, hemispheric asymmetry and cortical plasticity [78]. Future research should prioritize large-scale, longitudinal validation, multi-center clinical trials, and efforts to improve interpretability, so as to translate computational advances into reliable tools for precision psychiatry and affective neuroscience.

## Limitation

Despite a PRISMA-guided search across eight major databases, several limitations affect interpretation. First, only 34 studies were included, and relevant papers may have been missed due to database coverage, keyword choices, and the exclusion of some grey or non-indexed literature. Second, substantial variation in datasets, label schemes, preprocessing, and validation protocols limits cross-study comparability, so reported accuracies and standard deviations should not be treated as definitive rankings. Third, inconsistent reporting of steps such as windowing, artifact handling, and cross-subject splitting prevented a quantitative meta-analysis. Therefore, the findings are qualitative and trend-focused rather than effect size-based. Finally, most evidence comes from controlled benchmark datasets, so clinical translation remains uncertain until larger, multi-center, longitudinal evaluations are available.

## Conclusion

This study reviews recent advances in graph-based spatial attention mechanisms for EEG emotion recognition, with most work focusing on GCN- and GAT-based methods. In subject-dependent settings, many studies show performance improvements from dynamic graph construction and multidimensional attention. In subject-independent contexts, cognitive priors and domain adaptation are common and may enhance generalization, though results depend on specific protocols. Dataset composition, preprocessing choices, and validation strategies significantly influence outcomes and complicate comparisons. Overall, moving toward more adaptive graph models and integrating physiologically motivated constraints with data-driven learning, potentially boosting robustness and interpretability. Key challenges include improving cross-subject reliability and creating lightweight architectures with a better neurophysiological basis. Progress depends on standardized evaluation, more interpretable models, and interdisciplinary efforts to facilitate practical neuroscience and clinical use.

## Data Availability

No datasets were generated or analysed during the current study.

## Declarations

This paper was polished using Grammarly AI for minor improvements in the language and readability of the manuscript. The authors reviewed and edited the content as needed and take full responsibility for the content of the publication.
